# 2,3-Diphosphoglyceric Acid Alleviating Hypoxic-Ischemic Brain Damage through p38 MAPK Modulation

**DOI:** 10.3390/ijms25168877

**Published:** 2024-08-15

**Authors:** Jiawei Ni, Jing Zhao, Haocong Chen, Wenjuan Liu, Meini Le, Xirong Guo, Xiaohua Dong

**Affiliations:** Hongqiao International Institute of Medicine, Tongren Hospital, Shanghai Jiao Tong University School of Medicine, Shanghai 200336, China; njw3736@shtrhospital.com (J.N.); zjzy5612@163.com (J.Z.); chc4647@shtrhospital.com (H.C.); lwj1924@shtrhospital.com (W.L.); lmn2046@shtrhospital.com (M.L.)

**Keywords:** hypoxic-ischemic encephalopathy, 2,3-Diphosphoglyceric acid, OGD/R, neuronal apoptosis, MAPK

## Abstract

Neonatal hypoxic-ischemic encephalopathy (HIE) is a critical condition characterized by significant brain damage due to insufficient blood flow and oxygen delivery at birth, leading to high rates of neonatal mortality and long-term neurological deficits worldwide. 2,3-Diphosphoglyceric acid (2,3-DPG), a small molecule metabolite prevalent in erythrocytes, plays an important role in regulating oxygen delivery, but its potential neuroprotective role in hypoxic-ischemic brain damage (HIBD) has yet to be fully elucidated. Our research reveals that the administration of 2,3-DPG effectively reduces neuron damage caused by hypoxia-ischemia (HI) both in vitro and in vivo. We observed a notable decrease in HI-induced neuronal cell apoptosis, attributed to the downregulation of Bax and cleaved-caspase 3, alongside an upregulation of Bcl-2 expression. Furthermore, 2,3-DPG significantly alleviates oxidative stress and mitochondrial damage induced by oxygen-glucose deprivation/reperfusion (OGD/R). The administration of 2,3-DPG in rats subjected to HIBD resulted in a marked reduction in brain edema and infarct volume, achieved through the suppression of neuronal apoptosis and neuroinflammation. Using RNA-seq analysis, we validated that 2,3-DPG offers protection against neuronal apoptosis under HI conditions by modulating the p38 MAPK pathway. These insights indicated that 2,3-DPG might act as a promising novel therapeutic candidate for HIE.

## 1. Introduction

Neonatal hypoxic-ischemic encephalopathy (HIE), a condition characterized by brain damage resulting from inadequate blood flow and oxygen delivery to the brain around the time of birth, is a leading cause of neonatal death and long-term neurological disabilities globally [1,2]. The prevalence of HIE is estimated to range from 1.5 to 2.5 per 1000 live births in developed nations, with even higher rates observed in developing countries [3]. Therapeutic hypothermia, involving whole-body or head cooling, has emerged as the standard treatment for moderate to severe cases of HIE. Evidence shows that cooling within the first six hours post-birth can significantly decrease mortality and reduce the incidence of neurodevelopment disability in survivors. Nonetheless, therapeutic hypothermia’s effectiveness in mitigating neuronal damage does not exceed 50% [4,5]. Accumulating evidence highlights the potential benefits of erythropoietin and stem cell therapies for treating HIE; however, further clinical trials are essential to conclusively validate the effectiveness of these treatments [6,7,8]. This underscores the critical need for the development of novel neuroprotective strategies to enhance the overall efficacy of HIE neuroprotection.

Multiple cascades of events contribute to the brain damage observed in HIE, including oxidative stress, mitochondrial dysfunction, inflammation, and excitotoxicity, and ultimately lead to cell death [9]. Initially, hypoxic-ischemic (HI) conditions disrupt the energy supply, leading to a cellular environment imbalance. This disruption in energy metabolism then prompts an abnormal rise in intracellular calcium ion concentration, instigating cell damage [10]. Additionally, HI conditions provoke oxidative stress, generating a significant number of free radicals that further intensify cell damage and activate apoptosis signaling pathways. A critical aspect of this process is the imbalance between pro-apoptotic proteins, like Bax, and anti-apoptotic proteins, such as Bcl-2. This imbalance increases mitochondrial membrane permeability and results in the release of apoptogenic factors, including cytochrome c, ultimately triggering the activation of caspase family proteases, notably caspase-3, and facilitating cell apoptosis. Research has indicated that pathways such as MAPK, PI3K/AKT, and AMPK/Sirt1 are involved in HI-induced neuronal apoptosis [11,12,13]. Effectively inhibiting apoptosis has been shown to significantly mitigate HI-mediated brain injury.

Small molecules are increasingly being recognized as pivotal in the development of new therapies for infectious diseases, neurological conditions, and cardiovascular disorders. It has been recently highlighted that the 245 drugs approved by the U.S. FDA are either small or macromolecules [14]. The role of small molecule metabolites extends beyond their function as crucial intermediates in intracellular biochemical processes, and also serve as “signaling molecules” that are released into the extracellular space, influencing the target cells [15,16]. It was reported that the indole-3 propionate plays a role in axonal regeneration and the restoration of sensory function [17]. Furthermore, *N*,*N*-dimethylglycine has been shown to facilitate peripheral nerve regeneration following injury [18]. However, whether other endogenous metabolites are involved in neural damage repair remains to be determined.

2,3-Diphosphoglyceric acid (2,3-DPG) is a biochemically significant molecule, predominantly in human erythrocytes. It critically regulates the release of oxygen from hemoglobin to tissues [19]. As a byproduct of glycolysis in red blood cells, it forms from 1,3-bisphosphoglycerate through bisphosphoglycerate mutase and is broken down by bisphosphoglycerate phosphatase [20]. The main function of 2,3-DPG is to bind with deoxyhemoglobin, reducing hemoglobin’s oxygen affinity, thereby optimizing oxygen delivery, particularly during physical exertion or hypoxia [21,22,23]. Adaptively, the body elevates 2,3-DPG levels in red blood cells in high-altitude or chronic lung disease scenarios, enhancing tissue oxygenation [24]. While 2,3-DPG’s modulation of the oxygen-hemoglobin dissociation curve is vital for oxygen delivery in hypoxic conditions [25,26], its specific role in hypoxic-ischemic encephalopathy (HIE) remains under investigation. In this study, we found that 2,3-DPG mitigates neuronal damage induced by HI by modulating the p38 MAPK pathway both in vitro and in vivo, leading to a reduction in neuronal apoptosis. This insight positions 2,3-DPG as a promising new agent for the development of therapeutic drugs aimed at treating HIE.

## 2. Results

### 2.1. 2,3-Diphosphoglyceric Acid Mitigates HI-Induced Brain Damage through Regulated MAPK Signaling

#### 2.1.1. 2,3-Diphosphoglyceric Acid Reduced the Neuronal Cell Death Induce by OGD/R

**Figure 1:**
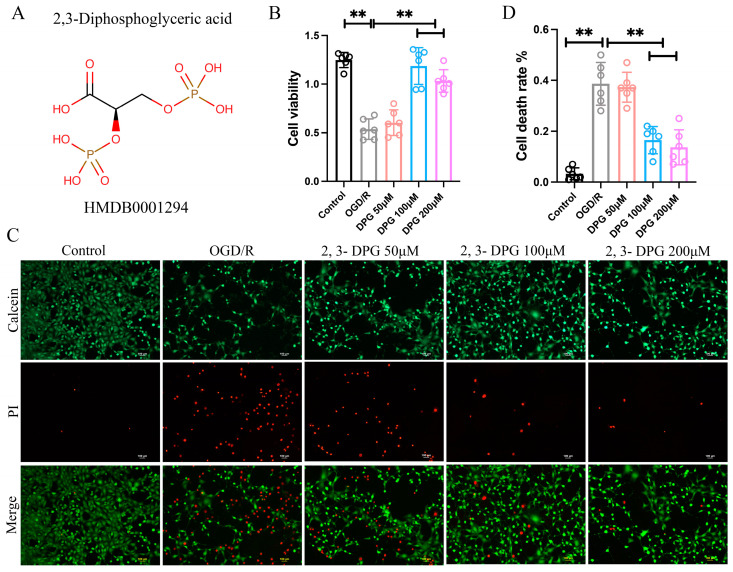
Inhibition of OGD/R-induced HT22 cell death by 2,3-Diphosphoglyceric acid (2,3-DPG). (**A**) Depicts the molecular structure of 2,3-Diphosphoglyceric acid (HMDB0001294). (**B**) The cell viability of control, OGD/R treatment, and OGD/R treatment with varying concentrations (50 μM, 100 μM, and 200 μM) of 2,3-DPG on HT22 (*n* = 6/group). The experiments were repeated three times, with 6 replicates for each group in each experiment. (**C**) Calcein/PI staining of HT22 cells under control conditions, OGD/R treatment, and OGD/R treatment supplemented with 50 μM, 100 μM, and 200 μM 2,3-DPG. The experiments were repeated three times, with 6 fields of view analyzed for each group in each experiment. (**D**) Quantitatively analyzes the cell death rate as indicated in C (*n* = 6/group). Data are expressed as mean ± SD. ** *p* < 0.01.

To explore the involvement of 2,3-Diphosphoglyceric acid (Figure 1A) in the pathogenesis of neuronal damage caused by OGD/R, we established the OGD/R-induced HT22 cell model and treatment with 2,3-Diphosphoglyceric acid. The results demonstrated a significant reduction in the viability of HT22 cells following OGD/R treatment. Conversely, there was a remarkable increase in viability observed in the OGD/R group treated with 100 μM and 200 μM of 2,3-Diphosphoglyceric acid (Figure 1B). In addition, calcein-AM/PI staining showed that the percentage of PI-positive cells in the OGD/R group was dramatically increased compared with that in the control group, but the dead cells were significantly decreased in 2,3-Diphosphoglyceric acid treatment group compared with the OGD/R group (Figure 1C,D). The result indicated that 2,3-Diphosphoglyceric acid alleviate OGD/R induced neuronal cell death.

#### 2.1.2. 2,3-Diphosphoglyceric Acid Inhibited HI-Induced Apoptosis In Vitro

**Figure 2:**
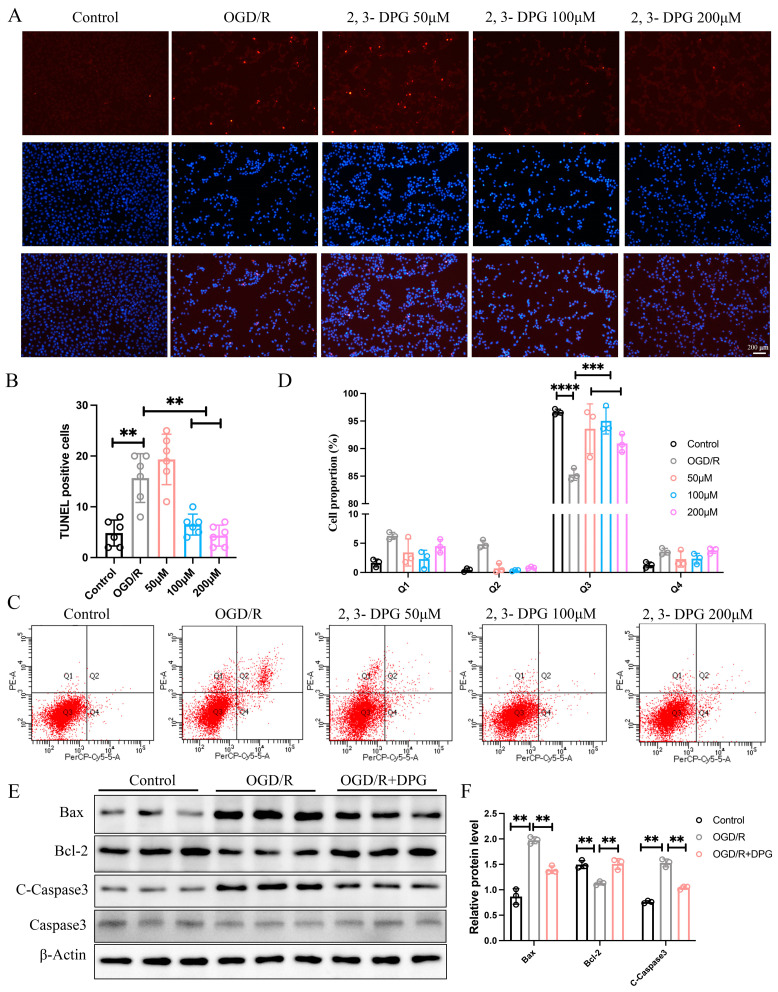
2,3-DPG suppressed the OGD/R-induced cell apoptosis. (**A**) Representative fluorescence graphs displaying cell apoptosis in control, OGD/R, OGD/R with 50, 100, and 200 μM 2,3-DPG treatments. The experiments were repeated three times, with 6 fields of view analyzed for each group. (**B**) Quantitative analysis of TUNEL-positive cells in A (*n* = 6/group). (**C**) Flow cytometry analysis was performed using Annexin V-FITC/PI staining to detect cell apoptosis in control, OGD/R only, and OGD/R treated with 50, 100, and 200 μM 2,3-DPG in HT22 cells. (**D**) Quantitative analysis of cell proportions in each quadrant of C (*n* = 3/group). The experiments were repeated three times, with 3 replicates for each group in each experiment. (**E**) Western blot analysis shows the expression levels of apoptotic markers Bax, Bcl-2, cleaved Caspase-3 (C-Caspase3), and Caspase3 with β-Actin as a loading control across control, OGD/R, and OGD/R with 200 μM 2,3-DPG treatment group. The total protein for the western blot analysis was obtained from three separate and independently repeated experiments. (**F**) Quantification of Bax, Bcl-2, and cleaved Caspase-3 protein levels in E (*n* = 3/group). Data represented as mean ± SD; ** *p* < 0.01, *** *p* < 0.001, **** *p* < 0.0001.

To explore whether 2,3-Diphosphoglyceric acid ameliorated OGD/R-induced neuronal cell injury by suppressed the apoptosis, TUNEL assays were used to detect the HT22 cell apoptosis in control, OGD/R treatment, and OGD/R treatment supplemented with the 2,3-Diphosphoglyceric acid group. The results showed that the apoptosis of HT22 cells was dramatically increased in OGD/R-induced HT22. However, in the 2,3-Diphosphoglyceric acid-treated OGD/R group, the number of apoptosis cells was significantly decreased (Figure 2A,B). Moreover, a flow cytometry assay revealed that the late apoptotic cells were significantly decreased in the 2,3-Diphosphoglyceric acid treatment group compared with the OGD/R group (Figure 2C,D). In addition, western blot results showed that the protein expression of Bax and cleaved-caspase 3 were all elevated in the OGD/R treatment group compared with the control group, while the expression of Bcl-2 was dramatically decreased in the OGD/R treatment group compared with the control group (Figure 2E,F). However, in the 2,3-Diphosphoglyceric acid-treated OGD/R group, the expression of Bax and cleaved-caspase 3 were dramatically decreased, and the expression of Bcl-2 was significantly increased (Figure 2E,F). The expression of total Caspase 3 remained unchanged in both the OGD/R and 2,3-Diphosphoglyceric acid treatment groups (Figure 2E). These results suggest that 2,3-Diphosphoglyceric acid exhibits neuroprotective effects against OGD/R-induced apoptosis in HT22 cells.

#### 2.1.3. 2,3-Diphosphoglyceric Acid Suppressed the OGD/R-Induced ROS Generation and Alleviates OGD/R-Mediated Mitochondria Damage

**Figure 3:**
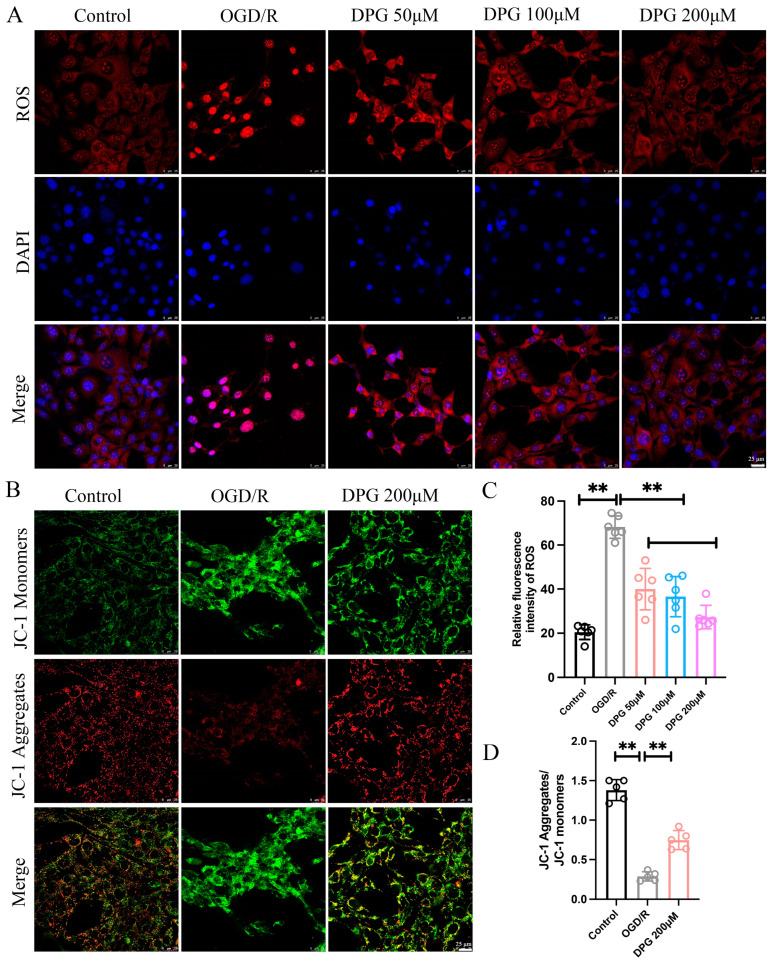
Impact of 2,3-DPG on oxidative stress and mitochondrial membrane potential in HT22 cells. (**A**) Fluorescence imaging of reactive oxygen species (ROS) production in control, OGD/R, and OGD/R with 2,3-DPG (50 μM, 100 μM, 200 μM) treatments. The experiments were repeated three times, with 6 fields of view analyzed for each group. Scale bar, 25 μm. (**B**) JC-1 staining of mitochondrial membrane potential in control, OGD/R, and OGD/R treated with 200 μM 2,3-DPG. Green fluorescence represents mitochondrial membrane depolarization, red fluorescence indicates mitochondrial membrane hyperpolarization. The experiments were repeated three times, with 6 fields of view analyzed for each group. Scale bar, 25 μm. (**C**) Quantification of ROS intensity levels in A (*n* = 6/group). (**D**) Ratio of JC-1 aggregates to monomers in B (*n* = 3/group). Data represented as mean ± SD; ** *p* < 0.01.

Oxidative stress plays a vital role in HIE-induced neuronal apoptosis. It was reported that 2,3-Diphosphoglyceric acid could reduce the ROS. To investigate whether 2,3-Diphosphoglyceric acid reduced the apoptosis of HT22 by inhibiting oxidative stress, the intracellular levels of ROS and mitochondrial membrane potential were measured. The results showed that the 2,3-Diphosphoglyceric acid administrated remarkedly decreased the OGD/R-induced ROS accumulation in HT22 cells (Figure 3A,B). In addition, the mitochondrial staining using JC-1 revealed that 2,3-Diphosphoglyceric acid treatment significantly elevated the mitochondrial activity in OGD/R-induced HT22 cells (Figure 3C,D). The result indicated that 2,3-Diphosphoglyceric acid suppressed the OGD/R-induced ROS accumulation and mitigated OGD/R-mediated mitochondrial damage.

#### 2.1.4. 2,3-Diphosphoglyceric Acid Treatment Ameliorated HI-Induced Brain Injury in Neonatal HIBD Rats

**Figure 4:**
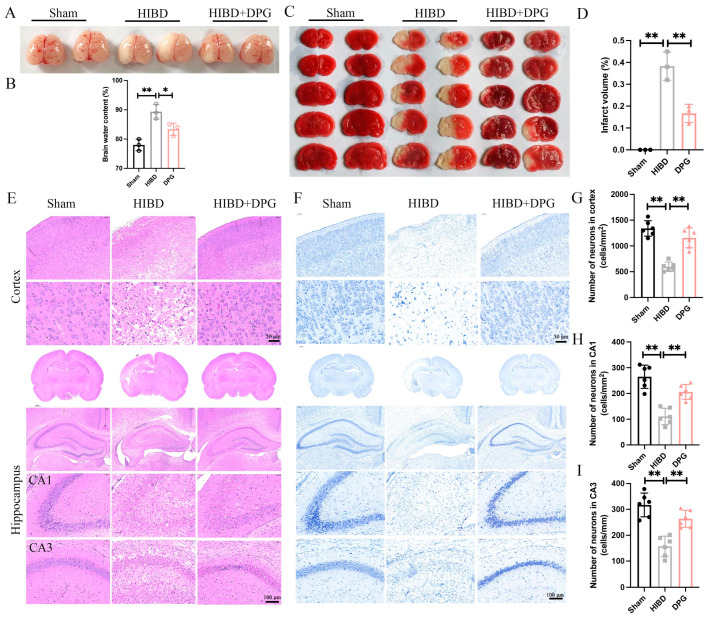
2,3-DPG reduces the nerve damage in neonatal HIBD rats. (**A**) The morphology of cerebral edema in sham, HIBD, and 2,3-DPG intervention of HIBD group at 48 h after surgery. (**B**) Statistical analysis of the brain water content from A (*n* = 3/group). (**C**) TTC staining of the cerebral infarction in sham, HIBD, and 2,3-DPG intervention of HIBD group at 48 h after surgery. (**D**) Quantification of the infarct area in D (*n* = 3/group). HE staining (**E**) and Nissl staining (**F**) of the whole brain with enlarged cortex and hippocampus region in sham, HIBD, and 2,3-DPG treated with TA group. Scale bar, 50 μm or 100 μm. Six rats of each group were analyzed. Statistics analysis of the number of neurons (cells/mm^2^) in cortex (**G**), CA1 (**H**), and CA3 (**I**) regions in sham, HIBD, and HIBD treated with 2,3-DPG group (*n* = 6/group). Data are presented as mean with SEM, * *p* < 0.05, ** *p* < 0.01.

To investigate the protective effects of 2,3-Diphosphoglyceric acid against OGD/R-induced neuronal injury in vivo, a hypoxic-ischemic brain damage (HIBD) rat model was established. Post-surgery, HIBD rats received an intraventricular injection of 200 μM 2,3-Diphosphoglyceric acid, while the control group received a vehicle solution. The occurrence of brain edema was significantly reduced in the 2,3-Diphosphoglyceric acid-treated HIBD rats compared to the vehicle-treated group 48 h after surgery (Figure 4A,B). Additionally, a notable decrease in brain water content was observed in the 2,3-Diphosphoglyceric acid-treated HIBD rats, as depicted in (Figure 4C,D). TTC staining revealed a substantial reduction in brain infarct volume in the 2,3-Diphosphoglyceric acid-treated group compared to untreated HIBD rats (Figure 4E,F). Histological analysis using Hematoxylin and Eosin (HE) and Nissl staining illustrated that the sham group’s cortex and hippocampus regions had well-organized neurons with clear structures and round nucleoli. In contrast, the HIBD group exhibited loosely arranged tissue with significant neuronal damage, including obvious nuclear contraction and abnormal morphology in the cortex and hippocampus (Figure 4E,F). Post-treatment with 2,3-Diphosphoglyceric acid significantly improved the arrangement of cells and morphology of neurons in the injured brain regions (Figure 4E,F). Furthermore, the quantification of neurons in the cortex, CA1, and CA3 regions of the hippocampus showed a dramatic decrease in neuronal numbers in the HIBD group compared to the sham group. However, neuronal numbers significantly recovered in the 2,3-Diphosphoglyceric acid-treated group (Figure 4G–I). These findings suggest that TA significantly mitigates neuronal damage in rats with HIBD.

#### 2.1.5. 2,3-Diphosphoglyceric Acid Inhibited HI-Induced Neuronal Apoptosis In Vivo

**Figure 5:**
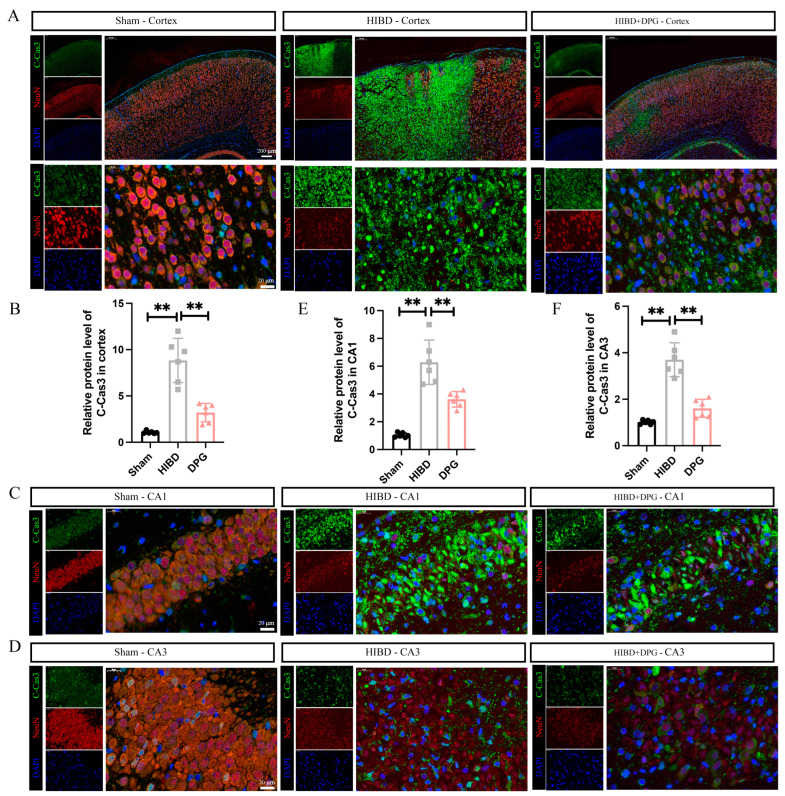
2,3-DPG intervention alleviates HI-induced neuronal apoptosis in HIBD rats. (**A**) Immunofluorescence of cleaved-caspase 3 (in green) and NeuN (in red) in the cerebral cortex region and enlarged cerebral cortex in sham, HIBD, and 2,3-DPG-treated HIBD rats. Six rats of each group were analyzed. Scale bar, 200 μm or 20 μm. (**B**) Statistical analysis of the fluorescence area of cleaved-caspase 3 in cerebral cortex in A (*n* = 6/group). Immunofluorescence of cleaved-caspase 3 (in green) and NeuN (in red) in the CA1 hippocampus region (**C**) and CA3 hippocampus region (**D**) in sham, HIBD, and 2,3-DPG-treated HIBD rats; 6 rats of each group were analyzed. Scale bar, 20 μm. Statistical analysis of the fluorescence area of cleaved-caspase 3 in CA1 hippocampus region in C (**E**) and CA3 hippocampus region in D (**F**) (*n* = 6/group). Data are presented as mean with SEM. ** *p* < 0.01.

To assess whether 2,3-Diphosphoglyceric acid mitigates HI-induced neuronal death by suppressing apoptosis in vivo, double immunostaining for cleaved-caspase 3 and NeuN was conducted on brain tissues from sham, HIBD rats, and HIBD rats treated with 2,3-Diphosphoglyceric acid. The findings revealed a significant increase in cleaved-caspase 3 expression within the cortex and hippocampal CA1/CA3 regions in HIBD rats, indicating enhanced apoptosis. Conversely, treatment with 2,3-Diphosphoglyceric acid markedly decreased cleaved-caspase 3 levels in these regions among HIBD rats (Figure 5A–F). These observations indicate that 2,3-Diphosphoglyceric acid effectively counters HI-induced neuronal damage by inhibiting apoptotic pathways.

#### 2.1.6. 2,3-Diphosphoglyceric Acid Effectively Mitigated Neuroinflammation in Rats with HIBD

**Figure 6:**
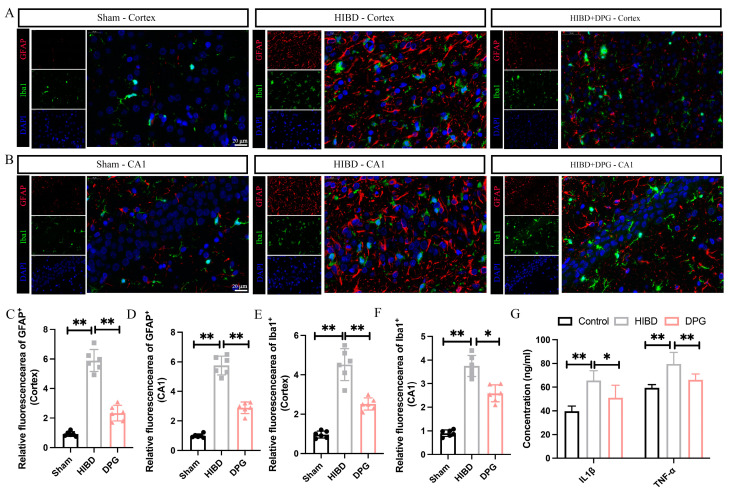
2,3-DPG intervention alleviates HI-induced neuroinflammation in HIBD rats. (**A**) Immunofluorescence of Iba1 (in green) and GFAP (in red) in the cerebral cortex region in sham, HIBD, and 2,3-DPG-treated HIBD rats; 6 rats of each group were analyzed. Scale bar, 20 μm. (**B**) Immunofluorescence of Iba1 (in green) and GFAP (in red) in the CA1 hippocampus region in sham, HIBD, and 2,3-DPG-treated HIBD rats; 6 rats of each group were analyzed. Statistical analysis of the fluorescence area of GFAP in cerebral cortex (**C**) and in cerebral cortex hippocampus (**D**) (*n* = 6/group). Statistical analysis of the fluorescence area of Iba1 in cerebral cortex (**E**) and in CA1 hippocampus region (**F**) (*n* = 6/group). (**G**) ELISA detection of IL-1β and TNF-α levels in rat brain tissue. (*n* = 6/group). Data are presented as mean with SEM. * *p* < 0.05, ** *p* < 0.01.

It was reported that neuroinflammation, triggered by damaged and dying neurons, exacerbates secondary neuronal damage and impairs brain functionality. To examine the potential anti-inflammatory effects of 2,3-Diphosphoglyceric acid in HI-induced rat models, the activation of microglia and astrocytes were detected via double immunostaining of the GFAP (an astrocytic marker) and Iba1 (a microglial marker) in the whole brain of sham, HIBD, and 2,3-Diphosphoglyceric acid-treated HIBD rats. The results revealed significant activation and proliferation of astrocytes in the cortex and CA1 region of the HIBD brain (Figure 6A,B). However, the proliferation and activation of astrocytes were significantly diminished following 2,3-Diphosphoglyceric acid intervention at 48 h post-HI, indicating a reduction in neuroinflammation (Figure 6A,B). Furthermore, the GFAP fluorescence area within the injured cortex and hippocampus was considerably larger in the HIBD group than in the sham group. However, the fluorescence area of GFAP significantly decreased after 2,3-Diphosphoglyceric acid treatment (Figure 6A–D). Consistently, HI-induced pronounced microglial activation in the HIBD group’s cortex and hippocampus, characterized by shorter, thicker branches and enlarged somas at 48 h post-HI (Figure 6A,B). However, 2,3-Diphosphoglyceric acid treatment markedly reduced microglial activation, with most Iba1+ cells returning to the morphological characteristics typical of their quiescent state, aligning with observations in the sham group (Figure 6A,B). This treatment significantly mitigated microglial activation in HIBD rats, demonstrating a reduction in both the number and the activation state of these cells compared to the HIBD group (Figure 6A,B,E,F). Furthermore, ELISA analysis revealed that the expression levels of IL-1β and TNF-α were significantly decreased in the brain tissues of HIBD rats treated with 2,3-Diphosphoglyceric acid compared to untreated HIBD rats (Figure 6G). These findings indicate that 2,3-Diphosphoglyceric acid effectively modulates the proinflammatory response triggered by HI in rats with HIBD.

#### 2.1.7. Differentially Expressed Signaling Pathways Involved in OGD/R-Induced Neuronal Injury

**Figure 7:**
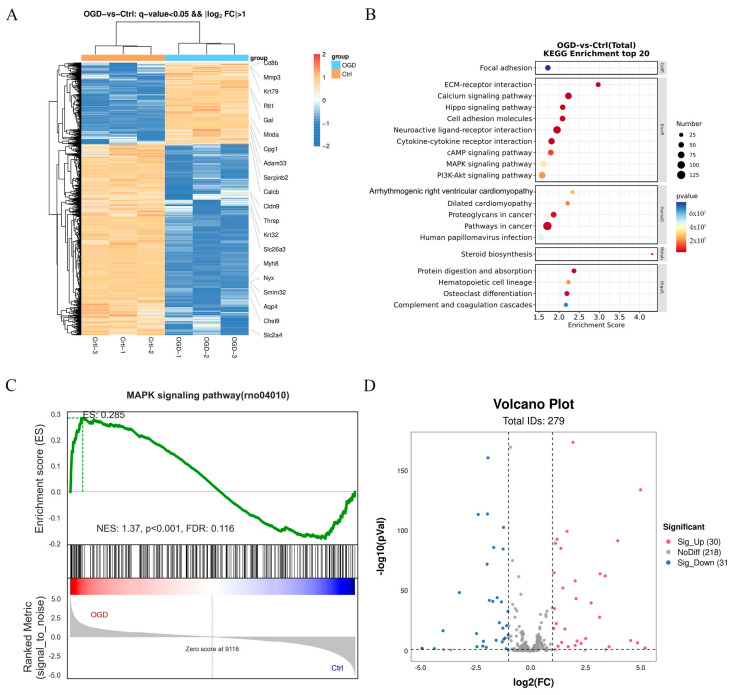
RNA-seq analysis revealed the MAPK signaling activation involved in the neuron damage in HT22. (**A**) Heat map displaying changes in differentially expressed mRNAs in OGD/R-induced HT22 compared with controls. (**B**) Top 20 enriched KEGG pathway of differentially expressed mRNAs. (**C**) GSEA overview of MAPK signaling in OGD/R-induced HT22 compared with controls. (**D**) Volcano plots of differentially expressed mRNAs of MAPK signaling pathway between OGD/R-induced HT22 and relative controls. The blue and red points in the plot represents the downregulated and upregulated expressed mRNAs with statistical significance, respectively.

To investigate the mechanisms by which 2,3-Diphosphoglyceric acid exerts its anti-apoptotic effects, RNA sequencing was conducted on HT22 cells subjected to oxygen-glucose deprivation/reoxygenation (OGD/R) and on control cells. The sequencing analysis revealed a total of 3020 differentially expressed mRNAs (*p* < 0.05, |log2 fold change| > 1) between the OGD/R-treated group and the control group. Specifically, 904 mRNAs were upregulated and 2116 were downregulated. Further analysis through the KEGG pathway elucidated that MAPK signaling was implicated in the neuronal damage induced by OGD/R, which was consistent with what was previously reported (Figure 7B). GSEA (Gene Set Enrichment Analysis) analysis found that the differently expressed genes were positively correlated with MAPK signaling, which including 30 upregulated and 31 downregulated genes (Figure 7C,D). The results indicated that MAPK signaling might participate in the anti-apoptosis role of 2,3-Diphosphoglyceric acid.

#### 2.1.8. p38 MAPK Signaling Pathway Involved in the Neuroprotective Effect of 2,3-Diphosphoglyceric Acid

**Figure 8:**
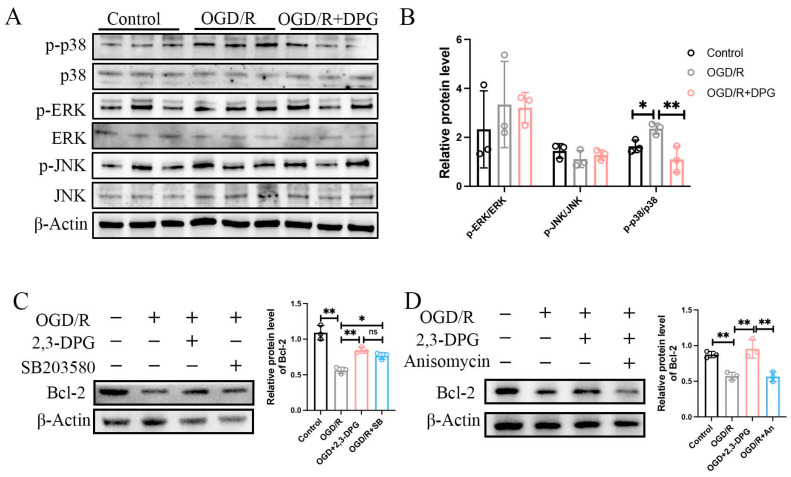
p38 MAKP signaling involved in the neuroprotection role of 2,3-DPG. (**A**) Western blot analysis of ERK, JNK and p38 MAPK signaling in control, OGD/R, and OGD/R with 200 μM 2,3-DPG treatment group. (**B**) Statistical analysis of the expression of p-ERK/ERK, p-JNK/JNK and p-p38/p38 in (**A**) (*n* = 3/group). (**C**) Western blot analysis and statistical analysis of Bcl-2 in control, OGD/R, OGD/R with 200 μM 2,3-DPG treatment group, and OGD/R with 10 μM SB203580 group (*n* = 3/group). (**D**) Western blot analysis and statistical analysis of Bcl-2 in control, OGD/R, OGD/R with 200 μM 2,3-DPG treatment group, and OGD/R combine treatment with 200 μM 2,3-DPG and 50 μM Anisomycin group (*n* = 3/group). Data represented as mean ± SD; * *p* < 0.05, ** *p* < 0.01; ns, no significance.

To verify whether 2,3-Diphosphoglyceric acid modulates cellular apoptosis through the MAPK signaling pathway, we analyzed the expression levels of MAPKs in HT22 cells subjected to OGD/R and treated with 2,3-Diphosphoglyceric acid by preformed western blot. The results show that OGD/R treatment did not significantly alter the levels of p-ERK/ERK or p-JNK/JNK. However, a notable increase in the ratio of p-p38 MAPKs to p38 MAPKs was observed in HT22 cells following OGD/R induction. Notably, the increase in p-p38/p38 was significantly reduced in cells treated with 2,3-Diphosphoglyceric acid, indicating a downregulation of p-p38 MAPK activity (Figure 8A,B). Additionally, Western blot analysis revealed that treatment with the p38 inhibitor SB203580 increased the OGD/R-induced downregulation of Bcl-2 in HT22 cells (Figure 8C). Moreover, the p38 agonist abolished the 2,3-Diphosphoglyceric acid-induced upregulation of Bcl-2 expression in OGD/R-treated HT22 cells (Figure 8D). These findings suggest that 2,3-Diphosphoglyceric acid might plays a crucial role in promoting neuronal survival by modulating p38 MAPK signaling.

## 3. Discussion

HIE remains a significant cause of mortality and neurodevelopmental impairment in newborns, with an ongoing absence of efficacious pharmacological treatments. In this study, we explored the effects of 2,3-diphosphoglycerate (2,3-DPG) on neuronal injury induced by oxygen-glucose deprivation/reperfusion (OGD/R) and investigated its underlying molecular mechanisms. Our findings demonstrate that 2,3-DPG significantly reduces neuronal cell death caused by OGD/R treatment in HT22 cells. Notably, 2,3-DPG treatment resulted in the modulation of key apoptotic markers, including a reduction in the expression of Bax and cleaved-caspase 3 and an increase in Bcl-2 expression. Moreover, our study highlights the role of 2,3-DPG in mitigating oxidative stress and mitochondrial damage induced by OGD/R. Furthermore, administering 2,3-DPG to rats with HIBD effectively diminished brain edema and infarct volume by suppressing neuronal apoptosis and neuroinflammation. These results suggest that 2,3-DPG effectively ameliorates HI-induced brain injury, offering promising therapeutic potential.

The glycolytic intermediate metabolites, including glucose-6-phosphate, fructose-6-phosphate, fructose-1,6-bisphosphate were upregulated in brain tissues at 6 h after cerebral ischemia of mice [27]. Conversely, in ischemic and preconditioned animals, a significant reduction in glycolytic intermediates such as lactate, pyruvate, and acetate has been observed [28]. Previous studies have highlighted the neuroprotective role of glycolytic intermediates, exemplified by the reduction of CNS injury in HIBD rats following treatment with fructose-1,6-bisphosphate [29]. Additionally, 2-phosphoglyceric acid has been found to mitigate HI-induced neuronal ferroptosis by regulating the GXP4/ACSL4 axis [30]. However, whether other glycolytic intermediate metabolites exert similar neuroprotective effects in the context of HI-induced neuronal injury remains unclear and warrants further investigation.

2,3-DPG, a crucial endogenous glycolytic metabolite, significantly influences the regulation of hemoglobin’s affinity for oxygen. Under normal physiological conditions, 2,3-DPG is found in high concentrations and is produced by red blood cells via the glycolytic pathway when exposed to hypoxic environments, thereby facilitating oxygen release [31,32]. It was reported that the levels of 2,3-DPG were reduced in diabetic neuropathy, and the reduced 2,3-DPG resulted in a defect in oxygen delivery within dorsal root ganglion neurons, followed by hypoxia and neuron death [33,34]. In addition, Jeffrey et al. suggested that elevated levels of 2,3-DPG and high-affinity hemoglobin in Parkinson’s disease may play a role in the oxidative damage that contributes to the neurodegenerative process [35]. However, the exact function of 2,3-DPG in neuronal damage associated with hypoxic-ischemic (HI) conditions remains unclear. In our present study, we found that 2,3-DPG significantly decreased oxidative stress and alleviated mitochondrial damage in OGD/R-mediated HT22 cells. However, how 2,3-DPG regulated the intracellular oxidative stress and mitochondrial functional recovery remains to be determined.

Neuronal apoptosis and neuroinflammation contribute to brain injury and are associated with the neuronal impairment and disability of newborns with HIE [1]. Previous studies have demonstrated increased expression of Bax and cleaved-caspase 3, and decreased Bcl-2 levels in both in vivo and in vitro models of HIE [36]. Consistent with these findings, our results revealed a significant activation of neuronal apoptosis in OGD/R-induced HT22 cells, confirmed by western blot and FACS analysis in HT22 cells, as well as immunofluorescence staining in the brains of HIBD rats. However, in the 2,3-DPG-treated OGD/R group and HIBD rats, the expression of activated caspase-3 was markedly decreased. Additionally, the inflammation response induced by HI was significantly reduced by inhibiting the activation of microglia and astrocytes in the brains of HIBD rats treated with 2,3-DPG.

MAPK signaling plays a vital role in regulating cell proliferation, differentiation, and apoptosis, and is essential for neuronal survival during HI-induced neuronal damage [11,37]. In mammals, there exist three prototypical subgroups of MAPKs, namely extracellular-regulated kinase (ERK1/2), c-Jun N-terminal kinase (JNK), and p38 MAPK [38]. Our previous research demonstrated the activation of JNK and p38 MAPK signaling in SHSY5Y cells under OGD/R conditions [11]. In the current study, we observed the activation of p38 MAPK in OGD/R-induced HT22 cells, and we found that intervention with 2,3-DPG inhibited the phosphorylation of p38. Furthermore, administering the p38 inhibitor increased the OGD/R-mediated reduction of Bcl-2 levels in HT22 cells, which indicated 2,3-DPG may reduce HI-induced neuronal damage by suppressing p38 MAPK signaling. However, our RNA-seq analysis revealed changes in other enriched KEGG pathways in OGD/R-induced HT22 cells compared to controls, including the calcium signaling pathway, cAMP signaling pathway, and PI3K-Akt signaling pathway. Further studies are warranted to elucidate the potential role of 2,3-DPG in these pathways.

In the current study, while we demonstrated the protective effects of 2,3-DPG against neuronal apoptosis induced by HI both in vitro and in vivo, an important limitation to consider is the role of 2,3-DPG as an energy-carrying metabolite. 2,3-DPG is metabolized by 2,3-bisphosphoglycerate phosphatase into 3-phosphoglycerate, which can re-enter the glycolytic pathway and contribute to ATP production [39]. Additionally, 3-phosphoglycerate is converted into 2-phosphoglycerate by phosphoglycerate mutase during the later stages of glycolysis [40,41]. Our previous research has shown that 2-phosphoglycerate also plays a neuroprotective role by combating HI-induced neuronal ferroptosis [30]. This raises the possibility that the observed beneficial effects of 2,3-DPG may not only be due to its specific molecular actions on p38 signaling pathways but also due to the supplementation of an energy source. Therefore, further studies are necessary to distinguish the effects of 2,3-DPG as a potential therapeutic agent from its role in energy metabolism.

## 4. Materials and Methods

### 4.1. Cell Culture and OGD Model

HT22 cells (mouse hippocampal neuron cells) were purchased from Wuhan Pricella Biotechnology Co., Ltd. (Wuhan, China) (CL-0697, RRID: CVCL_0321) and cultured in DMEM (Gibco, Carlsbad, CA, USA), supplemented with 10% fetal bovine serum, 1% penicillin, and 1% streptomycin (Gibco). The culture conditions were maintained at 37 °C in a humidified atmosphere containing 5% CO_2_. Oxygen-glucose deprivation (OGD) treatment was initiated when the cell confluency reached approximately 70%. For OGD/R treatment, the culture medium was replaced with glucose-free DMEM (Gibco), with 1% penicillin and 1% streptomycin. During OGD, HT22 cells were incubated in an environment of 5% CO_2_, 94.9% N_2_, and 0.1% O_2_ for 12 h. After OGD, HT22 cells were cultured with normal medium in a CO_2_ incubator for 12 h reoxygenation.

HT22 cells were subjected to treatment with 2,3-Diphosphoglyceric acid (D5764, Sigma, St. Louis, MO, USA) at concentrations of 50 μM, 100 μM, and 200 μM, respectively.

### 4.2. CCK8 Assay

Cell Counting Kit-8 (Beyotime, Shanghai, China) was used to evaluate the cell viability. Initially, the reaction solution was added to the culture medium at a 1/10 dilution ratio, followed by a 2-h incubation period at 37 °C with 5% CO_2_. Absorbance measurements were then taken at 450 nm to assess cell viability.

### 4.3. Calcein-PI Staining

Cell activity and cytotoxicity were assessed using the Calcein/PI Cell Viability/Cytotoxicity Assay Kit (Beyotime, Shanghai, China). Briefly, after washing the cells once with PBS, Calcein/PI working solution was added to cells. Subsequently, the cells were incubated for 30 min in an environment with 5% CO_2_ at 37 °C. The viability and cytotoxicity of the cells were then observed and analyzed under an inverted fluorescence microscope (Nikon, TS2-S-SM, Tokyo, Japan).

### 4.4. Flow Cytometry

The flow cytometry assay was performed as we reported previously [11]. Briefly, to detect apoptotic cells, fluorescence-activated cell sorting (FACS) using a BD LSRFortessa system, employed with an Annexin V-FITC/PI apoptosis detection kit (Vazyme, Nanjing, China). Initially, cells were enzymatically dissociated using 0.25% trypsin without EDTA. Subsequently, these cells were gathered via centrifugation at 1000 rpm for 3 min. Following centrifugation, cells were rinsed twice with chilled PBS under the same centrifugation conditions. Next, cells were resuspended in 100 μL of 1× binding buffer, to which 5 μL each of Annexin V-FITC and PI staining solutions were added. This mixture was then incubated for 10 min at room temperature. After incubation, an additional 400 μL of 1× binding buffer was added and the solution was mixed gently. Prior to analysis, the stained cells were passed through a 0.4 μm cell strainer (BD Falcon, Franklin Lakes, NJ, USA).

### 4.5. TUNEL Assay

Apoptosis in cells was assessed using the TUNEL assay kit (C1089, Beyotime, Shanghai, China), following the provided protocol. Briefly, cells were washed twice with PBS and then fixed with 4% paraformaldehyde (P0098, Beyotime, Shanghai, China) for 30 min. After fixation, the cells were again washed with PBS and then treated with 0.3% Triton X-100 in PBS for 5 min. After this permeabilization step, the cells underwent two additional PBS washes. The cells were then incubated with the TUNEL detection solution for 60 min. Following this incubation, cells were washed twice more with PBS. DAPI staining was then applied for 15 min, followed by another two PBS washes. Finally, the stained cells were visualized and imaged using a Nikon Eclipse Ts2-FL microscope (Nikon, Tokyo, Japan).

### 4.6. p38 MAPK Signaling Inhibition and Activation

To further verify the effect of p38 MAPK signaling on apoptosis, the p38 inhibitor, SB203580 (Beyotime, S1863, China), and the p38 activator, Anisomycin (MCE, HY-18982, Monmouth Junction, NJ, USA) was used to suppress and promote the p38 expression level. 10 μM SB203580 and 50 μM Anisomycin were used in OGD-induced HT22.

### 4.7. ELISA Assay

The levels of inflammatory factors in the cerebral cortex were measured according to the manufacturer’s instructions. A rat IL-1β (Interleukin 1 Beta) ELISA Kit (Beyotime, Shanghai, China) and a rat TNF-α ELISA Kit (Beyotime, Shanghai, China) were used.

### 4.8. Reactive Oxygen Species (ROS) Assay

Intracellular reactive oxygen species (ROS) levels were measured using a DHE-based ROS assay kit (C13002, Applygene Technologies Inc., Beijing, China), according to the manufacturer’s guidelines. Initially, the culture medium was removed from the cells. A 10 μM DHE probe solution, prepared by diluting the probe in serum-free culture medium, was then added to each sample. The cells were incubated with this solution for 30 min in a cell culture incubator maintained at 37 °C. After incubation, the supernatant was discarded, and the cells were rinsed twice with PBS. The fluorescence intensity, indicative of ROS production, was immediately assessed using a Lecia SP8 confocal microscope (Wetzlar, Germany).

### 4.9. Western Blot

HT22 cells were harvested using a RIPA (Radio Immunoprecipitation Assay) lysis buffer (Beyotime, Shanghai, China), supplemented with 1 mM PMSF (Phenylmethanesulfonyl fluoride) (Beyotime, Shanghai, China). Protein separation was performed using 8–20% acrylamide/bisacrylamide gels, followed by the transfer of proteins onto PVDF membranes (Millipore, Billerica, MA, USA). To block nonspecific protein binding, 5% blocking reagent (1 × TBST containing 5% defatted milk powder) was applied for 1 h at room temperature. The membranes were then incubated with primary antibodies (diluted at 1:1000–1:5000) in blocking reagent overnight at 4 °C. Subsequently, the membranes were washed three times with 1 × TBST and then incubated with HRP (horseradish peroxidase)-conjugated secondary antibodies (diluted at 1:1000–1:5000) in blocking reagent for 1 h at room temperature. The protein expression was detected using Tanon 6200 equipment (Tanon, Shanghai, China).

The following primary antibodies were utilized: Phospho-p38 MAPK (Thr180/Tyr182) (4511 T, Cell Signaling Technology, Danvers, MA, USA), p38 MAPK (66234-1-Ig, Proteintech, Wuhan, China), Phospho-JNK (Tyr185) (80024-1-RR, Proteintech, China), JNK (66210-1-Ig, Proteintech, China), Phospho-ERK1/2 (Thr202/Tyr204) (28733-1-AP, Proteintech, China), ERK1/2 (16443-1-AP, Proteintech, China), Phospho-JNK (Tyr185) (80024-1-RR, Proteintech, Wuhan, China), JNK (66210-1-Ig, Proteintech, China), cleaved-caspase 3 (Asp175) (#9661, Cell Signaling Technology), Bax (50599-2-Ig, Proteintech), Bcl-2 (#BF9103, Affinity, Changzhou, China), and beta actin mouse monoclonal antibody (66009-1-Ig, Proteintech, China). The secondary antibodies used were HRP-conjugated Affinipure Goat Anti-Mouse IgG (H + L) (SA00001-1, Proteintech, China) and Anti-Rabbit IgG (H + L) (SA00001-2, Proteintech, China).

### 4.10. Mitochondrial Membrane Potential (MMP) Measurement

The mitochondrial membrane potential (MMP) was assessed using the Mitochondrial Membrane Potential Assay Kit, which utilized JC-1 (5,5′,6,6′-tetrachloro1,1′,3,3′-tetramethylbenzimidazolylcarbocyanine iodide) for MMP detection, following the provided protocol. The MMP of HT22 was detected using 10 μg/mL JC-1. Confocal microscopy (Leica, SP8, Wetzlar, Germany) was immediately employed to capture the fluorescence intensity of JC-1 monomers (green fluorescence) and JC-1 aggregates (red fluorescence). Image J software (Version: 2.3.0) was utilized to quantify the fluorescence intensity of JC-1 monomers and JC-1 aggregates. The assessment of MMP was based on the ratio of red fluorescence to green fluorescence.

### 4.11. Immunofluorescence (IF)

Immunofluorescence (IF) was conducted following our previously published protocol [42]. HT22 cells and brain sections were fixed with 4% paraformaldehyde (PFA) at 4 °C for 20 min. Subsequently, the samples were incubated with NeuN antibody (#94403, Cell Signaling Technology), cleaved-caspase 3 antibody (#9661, Cell Signaling Technology), GFAP (#3670, Cell Signaling Technology), and Iba1 (#17198, Cell Signaling Technology), followed by secondary antibodies (Proteintech, SA00013-1/2/3/4). Confocal microscopy (Leica, SP8, Germany) was used to capture the images.

### 4.12. Neonatal Hypoxic-Ischemic Brain Damage (HIBD) Rat Model

The animal experiment received approval from the ethical committee of Shanghai Tongren Hospital in accordance with established standards for animal use and care (A2023-058-01). A neonatal HIBD model was established based on a modified version of the Rice–Vannucci method [43]. The neonatal rats were divided into three groups: the sham group, the HIBD group, and the HIBD group treated with 200 μM 2,3-Diphosphoglyceric acid (Sigma, Darmstadt, Germany). In brief, on the 7th day after birth, double-layer ligation (using 7-0 thread) was performed on the left common carotid artery of newborn rats under a microscope. In the sham group, neonatal rats underwent exposure of the left common carotid artery without ligation. After the surgical procedure, the pups were returned to their mothers for a 2-h period. Subsequently, the neonatal rats were placed in a low-oxygen chamber with 8% oxygen and 92% nitrogen for 2 h at 37 °C. Afterward, the neonates were reunited with their mothers. For the 2,3-Diphosphoglyceric acid treatment group, intraventricular injection of 2,3-Diphosphoglyceric acid commenced immediately after the surgery. After a 48-h recovery period, the rats were anesthetized, and their brain tissues were collected for TTC staining, hematoxylin and eosin (HE) staining, Nissl staining, and immunofluorescence staining.

### 4.13. TTC Staining

TTC staining was conducted in accordance with previously published methods [30]. The 2% solution of 2,3,5-Triphenyltetrazolium chloride (TTC) staining solution (Coolaber, Beijing, China) was employed for this purpose. At 48 h post-HIBD, the rat’s brain was harvested and sectioned into 2 mm thick coronal slices. These brain sections were then subjected to staining with the 2% TTC solution for a duration of 10–30 min at 37 °C. After staining, non-ischemic necrotic areas appeared light red, while ischemic necrotic tissue appeared white. The brain infarct volume was subsequently analyzed using Image J software.

### 4.14. Hematoxylin-Eosin (HE) Staining

The brain tissues were initially fixed overnight in 4% paraformaldehyde solution. Subsequently, the fixed brains underwent a series of procedures, including dehydration with ethanol, rendering them transparent through xylene treatment, and finally embedding them in paraffin. Coronal sections, each measuring 5 μm in thickness, were then cut from the paraffin-embedded blocks. Following this, the sections were dewaxed using xylene, rehydrated using alcohol, and stained using hematoxylin-eosin. The resulting slices were observed using the Pannoramic Scan Pannoramic SCAN II system (3DHISTECH, Budapest, Hungary).

### 4.15. Nissl Staining

The paraffin-embedded brain slides were subjected to a 2-h incubation at 60 °C. Subsequently, the sections were dewaxed with xylene I and xylene II for 20 min each, followed by rehydration using ethanol. After three washes with distilled water, the slides were immersed and stained at a temperature of 50–60 °C for 40 min in a methyl violet staining solution, then rinsed with deionized water. Next, the slides were dehydrated using ethanol, cleared with xylene, and finally mounted with natural gum. The resulting slides were then captured using the Pannoramic Scan Pannoramic SCAN II system (3DHISTECH, Hungary).

### 4.16. RNA-Sequencing Analysis (RNA-Seq)

RNA sequencing was conducted in accordance with our previously described methods [11]. HT22 was subjected to OGD/R treatment and untreated, was harvested for RNA sequencing. The analysis of RNA-seq data was performed by OE Biotech Co., Ltd. (Shanghai, China). DESeq2 was employed for differential expression analysis, with the parameters for identifying significantly differentially expressed genes (DEGs) being a *q*-value of less than 0.05 and a fold change greater than 1.5 or less than 0.83. To uncover significantly enriched terms, enrichment analysis on DEGs was executed across KEGG pathways, Reactome, and WikiPathways using the R software (version 3.2.0).

### 4.17. Quantification and Statistical Analysis

Data analysis was performed using GraphPad Prism software (Version 9.0, GraphPad Software, La Jolla, CA, USA). The unpaired Student’s *t*-test was applied for comparing two groups, while one-way ANOVA was utilized for comparing multiple groups. Statistical significance was defined as *p* < 0.05, with * indicating *p* < 0.05, ** indicating *p* < 0.01. Error bars represent the standard deviation (SD).

## 5. Conclusions

In summary, our results suggest that 2,3-Diphosphoglyceric acid (2,3-DPG) acts as a neuroprotective factor, mitigating neuronal cell apoptosis induced by hypoxia. These findings contribute to the growing understanding of 2,3-DPG’s potential as a therapeutic agent for protecting neurons against hypoxic injury. 2,3-DPG effectively downregulated the HI-induced Bax and cleaved-caspase 3 and upregulated the expression of Bcl-2, thereby reducing cell apoptosis. In addition, 2,3-DPG inhibited the HI-induced neuroinflammation through the decreased expression of Iba1 and GFAP in the cortex and hippocampal region of HIBD rats. Mechanistically, 2,3-DPG promotes cell survival through the regulation of the p38 MAPK signaling pathway to alleviate HI-induced apoptosis. In conclusion, 2,3-DPG is a potential therapeutic target for HIE.

## Data Availability

The data presented in this study are available on request from the corresponding author due to privacy and ethical considerations.
